# A Case for Diagnosis

**Published:** 1908-02-08

**Authors:** 


### A CASE FOR DIAGNOSIS.

A girl, aged sixteen, came under observation for paretic symptoms, which are described below. She had always been a delicate child; she was pale and thin, and for years had been the subject of severe headaches. On her admission to hospital she complained of pains in the head, not fixed in their locality, and of some stiffness in the neck. There was complete paralysis of the left arm, with flaccidity, and with some wasting and flabbiness of the muscles. She was able to walk, but in so doing she dragged her left leg with a sweeping movement, such as is frequently seen in cases diagnosed as functional paresis of a lower limb. There was no paralysis of the face or tongue, nor of any cranial nerve.

The patient was of delicate frame. She complained of severe pain at the top of her head. The catamenial function had been suspended for six months. There was no vomiting, nor any constipation. The urine was natural, and nothing could be found wrong with the lungs, heart, or abdominal organs. There was no optic neuritis.

The palsy of the left arm was complete; that of the leg not complete, and yet considerable; for although the girl could manage to walk with some difficulty, and could move the leg slowly up and down as she lay in bed, she was unable to hold it up from the bed. The reflexes in the right leg were natural; in the left leg the knee-jerk was increased, and there was extensor plantar reflex, but no ankle clonus.

Sensibility in the left arm and leg was impaired, although a considerable amount of feeling was still left in the paralysed side. Sensibility was normal in the right arm and leg, and in both sides of the head.

On further examination, attention was arrested by a marked stiffness of the patient's neck, in consequence of which the head was drawn to the right side, and the face looked rather towards the left. There was a very rigid state of the right sterno-mastoid muscle, such as one commonly sees in cases of wry-neck. On the left side the neck presented considerable deep-seated swelling in the region of the uppermost cervical vertebræ. It was evident to the touch that this swelling was not due to any accumulation of fluid, nor were the integuments or muscles obviously diseased. They were stretched over the swelling, and it felt as if a thickened state of the ligamentous tissues could be made out through them. Owing to pain, and to the rigidity of the right sterno-mastoid muscle, the rotatory and other movements of the head were much impeded, those to the right existing only to a slight degree, and those to the left being seemingly limited by some mechanical hitch. The patient complained very much of pain in the upper part of the neck, both when pressure was made upon it and when it was moved, and sometimes even when nothing was done to it at all. It was especially painful at night, and the girl found it very difficult to obtain a position of ease in which to lay her head. Her nights were consequently much disturbed.

On being questioned as to the origin of this affection of the neck, she stated that it had begun, about six months before her admission, with stiffness and pain, the head being drawn to the right side. Soon afterwards it began to swell, and as the swelling increased, she suffered much more pain. The palsy of the arm appears to have come on quite gradually, and she was unable to fix the precise date of its commencement. She was certain that it had come on subsequently to the appearance of the swelling. She first noticed weakness of the leg a fortnight before her admission, and this had gradually but steadily increased ever since.

She attributed the pain and swelling of the neck to a cold, and denied that she had ever received a blow there.

For the next twenty-three days the patient manifested no additional symptom of importance. She continued to suffer much from pain in the neck and loss of rest. She was treated by iron and iodide of potassium; opiates at night; aperients when necessary; and counter irritation, and afterwards opiate applications, to the neck. A generous diet was allowed.

On the morning of the twenty-third day after admission she was found to have lost the use of the left leg completely, and she was totally unable to do anything for herself. She also complained of twitching on the left side of her body. On more careful examination it was noticed that she had complete paralysis, not only of left arm and left leg, but also of the intercostal and abdominal muscles of the left side, which remained perfectly motionless during respiration. The umbilicus at each expiration was distinctly drawn to the right side.

Sensibility was not more impaired in the paralysed limbs than on her admission. No difference could be observed between the two sides as regards temperature. The pulse, which hitherto had not exceeded 100, now rose to 120.

A consultation was held, in which the two questions that most called for an answer were: What is likely to be the outcome of the girl's trouble? and What is the most probable lesion present, as a cause for her symptoms?

The answer to these two questions will be given in our next issue.

